# DNA Biosensor Based on Double-Layer Discharge for the Detection of HPV Type 16

**DOI:** 10.3390/s19183956

**Published:** 2019-09-13

**Authors:** José R. Espinosa, Marisol Galván, Arturo S. Quiñones, Jorge L. Ayala, Sergio M. Durón

**Affiliations:** 1 Electrical Engineering Department, Autonomous University of Zacatecas, Col. Centro, Av. Ramón López Velarde 801. Zacatecas, Zacatecas C.P. 98000, Mexico; electropinky@hotmail.com; 2 Chemistry Department, Autonomous University of Zacatecas, Campus Siglo XXI, Edif. 6, Km 6 carr. Zacatecas-Guadalajara, Zacatecas C.P. 98160, Mexico; gavm001144@uaz.edu.mx (M.G.); arturochemistry.qfb@gmail.com (A.S.Q.); jayala69@uaz.edu.mx (J.L.A.)

**Keywords:** double layer discharge, potential relaxation, electrochemical HPV-16 DNA biosensor, DNA/Au electrode equivalent circuit

## Abstract

DNA electrochemical biosensors represent a feasible alternative for the diagnosis of different pathologies. In this work, the development of an electrochemical method for Human Papillomavirus-16 (HPV-16) sensing is reported based on potential relaxation measurements related to the discharge of a complex double layer of a DNA-modified gold electrode. The method used allows us to propose an equivalent circuit (EC) for a DNA/Au electrode, which was corroborated by electrochemical impedance spectroscopy (EIS) measurement. This model differs from the Randles circuit that is commonly used in double-layer simulations. The change in the potential relaxation and associated charge transfer resistance were used for sensing the DNA hybridization by using the redox pair Fe(CN)_6_^4-^/Fe(CN)_6_^3+^ as an electrochemical indicator. In order to determinate only the potential relaxation of the composed double layer, the faradic and double-layer current contributions were separated using a rectifier diode arrangement. A detection limit of 0.38 nM was obtained for the target HPV-16 DNA sequences. The biosensor showed a qualitative discrimination between a single-base mismatched sequence and the fully complementary HPV-16 DNA target. The results indicate that the discharge of the double-layer detection method can be used to develop an HPV DNA biosensor.

## 1. Introduction

The human papillomavirus (HPV) is one of the most common sexually transmitted infections, affecting the skin and mucous membranes. HPV has been detected in virtually all invasive cervical cancers and has been confirmed as the major cause of cervical cancer [1]. HPV is a group of non-enveloped, double-stranded DNA viruses composed of approximately 200 genotypes [2]. It has been shown that the 71.8% of worldwide invasive carcinomas are associated with high-risk HPV types (HR-HPV), mostly HPV-16 and HPV-18 [3]. The majority of human HR-HPV infections are cleared by the immune system within 1–2 years and are asymptomatic. However, persistent infection with HR-HPV can induce cellular and histological changes leading to cancer [1]. Indirect evidence of HPV infection can be obtained through physical examination by colposcopy, as well as cytological and histological changes that are detected by Pap screening or biopsy [4]. In addition, biopsies can be analyzed by nucleic acid hybridization to directly detect the presence of HPV DNA and to identify different types of HPV strains [5].

DNA analyses are based on the complementarity principle of nucleic acid strands, and the main molecular methods based on the hybridization of nucleic acid are the polymerase chain reaction (PCR) and hybrid capture 2 (HC2). There are many PCR protocols, but all require the amplification of a fragment of the DNA target using consensus primers, then the PCR products are visualized under UV light after electrophoresis or using fluorescence techniques [6,7]. HC2 is a commercial kit based on hybrid capture technology for the detection of 13 high-risk types of HPV DNA in cervical specimens. The target DNA hybridize with a specific HPV RNA probe cocktail that are captured onto the surface of a microplate well coated with antibodies specific for RNA–DNA hybrids. Immobilized hybrids are then reacted with alkaline phosphatase-conjugated antibodies specific for the RNA–DNA hybrids, and detected with a chemiluminescent substrate [8,9]. Although PCR and HC2 present high sensitivity and specificity, they are mainly used by research labs and/or specific health services because of the long time required and the complex protocols that usually demand specialized skills for the correctly operating the instruments and monitoring the compliance of methods [10].

Nowadays, a wide variety of DNA biosensors based on quartz crystal microbalance [11] and optical transducers [12,13,14] have been developed for the specific detection of HPV DNA. Compared to the PCR and HC2, these biosensors are dedicated to detecting DNA strains, avoiding prolonged experimentation processes and purification requirements [10]. However, these techniques are relatively complex, and require specialized and expensive instruments. 

In recent years, electrochemical biosensors used in the detection of HPV–DNA hybridization, have received considerable attention due to the advantages of low-cost instrumentation, simplicity, high sensitivity, possibility of microfabrication technologies and portability, making them excellent candidates for point-of-care DNA diagnostics [15,16,17,18]. In electrochemical biosensing, electrochemical impedance spectroscopy (EIS) has several advantages in comparison with other detection techniques, and it is commonly used to verify stability, reliability and ability to preserve the integrity of the measured analyte. EIS provides a powerful yet simple method for measuring changes in the bulk or interfacial properties of materials including modified surfaces that allow molecular recognition events. A major advantage of EIS is that detection can be performed label-free (i.e., the changes in the electrical properties of the electrode surface arise from a direct interaction with the target molecule) [19]. Although EIS provides these conveniences, an important drawback of its utilization in compact point-of-care sensors is the time required to obtain a complete impedance spectrum, which typically lasts 20–30 min, as well as the electronic architecture associated with “frequency response analyser” or “lock-in amplifier” systems.

On the other hand, EIS data must be examined accurately and should be fitted to an adequate and physical meaningful circuit model for calculation of impedance parameters of an electrochemical measurement system [20]. Equivalent circuits (ECs) thus play a fundamental role in the interpretation of biosensor performance, and must be constructed using the essential elements that represent the electrode surface characteristics of the HPV DNA biosensor [21]. In recent biosensor studies regarding HPV recognition, a simple Randles equivalent circuit has been used for fitting the experimental EIS data to different types of DNA-modified electrodes [22,23,24]. However, this circuit is not enough to accurately fit the full values of experimental impedance data obtained at the different AC-applied frequencies, and it is not able to completely characterize the detection of DNA hybridization.

In order to gain further understanding of the electrochemical behavior of DNA hybridization and its possible applications in the diagnostic field, this paper investigates an electrochemical sensor for the DNA detection of specific high-risk HPV16 sequences. The sensor uses a rapid and simple measurement method based on the relaxation potential of the DNA/Au complex electrode double layer. Furthermore, based on the experimental evidence we propose an alternative equivalent circuit that could be applied to DNA-modified electrodes. This circuit fits the EIS experimental data with high precision and suggests the presence of multiple electron transfer regions in DNA sequences.

## 2. Materials and Methods

### 2.1. Chemicals and Reagents

**Figure 1:**
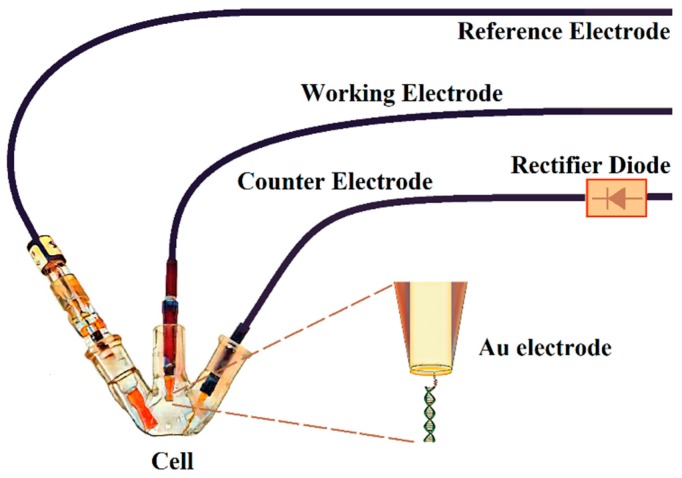
Schematic drawing of the electrochemical cell.

Electrochemical measurements were performed in a three-electrode system composed of a 0.5-cm radius Au disk with an immobilized layer of DNA probe as the working electrode (WE), a Hg/Hg_2_SO_4_/K_2_SO_4_ (sat) reference electrode and a Pt wire as a counter electrode. A schematic drawing of the used cell and general setup is shown in Figure 1. In this work, all reported potential values refer to the saturated sulfate reference electrode, except where indicated. The hybridization studies of bioelectrode were carried out using double potential step chronoamperometry and electrochemical impedance measurements made in 2 mM K_4_(Fe(CN)_6_) + 2 mM K_3_(Fe(CN)_6_) in 50 mM PBS+ 100 mM K_2_SO_4_ solution (pH 7.4) with an ionic strength of 447 mM. In each experiment, the solution was deoxygenated with high purity nitrogen for at least 5 min, and a nitrogen atmosphere was kept over the solution during electrochemical measurements. 

The oligonucleotide stock solutions were prepared with 20 mmol·L^−1^ Tris-HCl buffer, pH 7.4 solution (Tris) and kept frozen. The HPV-related 30-base oligonucleotide sequences used in the present study are shown in Table 1.

### 2.2. DNA Probe Immobilization and Hybridization with DNA Target

Gold disk working electrodes were polished with aluminum oxide particles of different sizes (1, 0.3 and 0.05 µm) to obtain a mirror surface. The electrodes were washed with water and sonicated for 15 min in isopropanol to remove any particles. Electrodes were subsequently electrochemically cleaned in 0.5 M H_2_SO_4_ by scanning the potential between −0.05 and +1.1 V for approximately 60 cycles until no further change in the voltammogram was obtained. 

The immobilization of probe DNA on the gold surface was performed by exposing the electrode to 20 µL of a solution of 1 µM single strand deoxyribonucleic acid (ssDNA) in immobilization buffer for 1 h at room temperature. The DNA immobilization buffer consisted of 0.8 M phosphate buffer (PB) + 1.0 M NaCl + 5 mM MgCl_2_ + 1 mM EDTA, pH 7.0. After immobilization, the electrode was sequentially rinsed in the following solutions: immobilization buffer, 200 mM PB, 10 mM PB and, finally, 10 mM PB + 10 mM EDTA to remove any remaining magnesium ion. To ensure complete thiol coverage of the gold surface and to avoid non-specific interactions of oligonucleotides, the electrodes were subsequently electrochemically cleaned by scanning the potential between the oxidation and reduction of the Fe(CN)_6_^4-^/Fe(CN)_6_^3^ redox couple, −0.4 and +0.4 V, for approximately 50 cycles until no further change was observed. 

For hybridization experiments the Au/ssDNA probe electrode was incubated in a 1-µM solution of complementary target DNA in PBS, pH = 7.4, for 1 h (90 °C). Finally, it was rinsed with 50 mM PB + 100 mM K_2_SO_4_, pH 7.0. The DNA immobilization and hybridization were analyzed using the Fe(CN)_6_^4-^/Fe(CN)_6_^3-^ redox couple. 

The experimental conditions for immobilization and hybridization were previously optimized. In this sense, the used concentration of ssDNA and immobilization time resulted in a surface concentration Γ = 2 × 10^12^ molecule cm^−2^ as estimated by chronocoulometry measurements. On the other hand, different times were essayed in hybridization optimization tests and no further changes were observed for times greater than 1 h. A high reproducibility was obtained when the reported conditions were used.

Biosensing response characteristics of DNA bioelectrode were studied after hybridization with a complementary target at concentrations of 1000, 100, 10 and 1 nM at room temperature. Limit of detection (LOD) was calculated as three times the standard deviation of the blank sample measurement; the blank was an Au/ssDNA electrode incubated at the same conditions described above but using a PBS solution without the complementary target DNA. For specificity assay solutions of complementary and single-base mismatch sequences were used, and the hybridization response of each one was compared by using a Student’s *t*-test.

### 2.3. Electrochemical Measurements

Electrochemical detection was performed by applying a double potential step from the open circuit potential (OCP) to a potential of 30 mV with respect to the OCP value during 750 µs. Then, the system was returned back to the OCP and the voltage discharging curves were registered for 25 ms. From these discharging curves, the potential relaxation was measured and the charge transfer resistance ($R_{ct}$) through the DNA-modified electrode was calculated by the method described in the next section. The presence of a complementary sequence was detected by the change in resistance when the ssDNA was transformed in double strand deoxyribonucleic acid (dsDNA) due to the hybridization event.

In order to corroborate the $R_{ct}$ values obtained from the relaxation curves, electrochemical measurements of the modified electrodes were performed in PBS solution (pH 7.0) by using the EIS technique and obtaining the resistance values by a non-linear least squares fitting (CNLS) of the experimental impedance data. The impedance was measured over the frequency range from 100 kHz to 100 mHz, with a 10 mV AC amplitude voltage superimposed on a DC bias of 30 mV with respect to the open circuit potential, which corresponds with the formal potential of the Fe(CN)_6_^4-^/Fe(CN)_6_^3-^ redox couple. The $R_{ct}$ value was measured before and after DNA hybridization. The solution resistance ($R_{s}$) was measured using the same technique with an excitation frequency of 100 kHz. The potential relaxation experiments and EIS were carried out using a Reference 600 Gamry potentiostat.

### 2.4. The Potential Relaxation Method 

**Figure 2:**
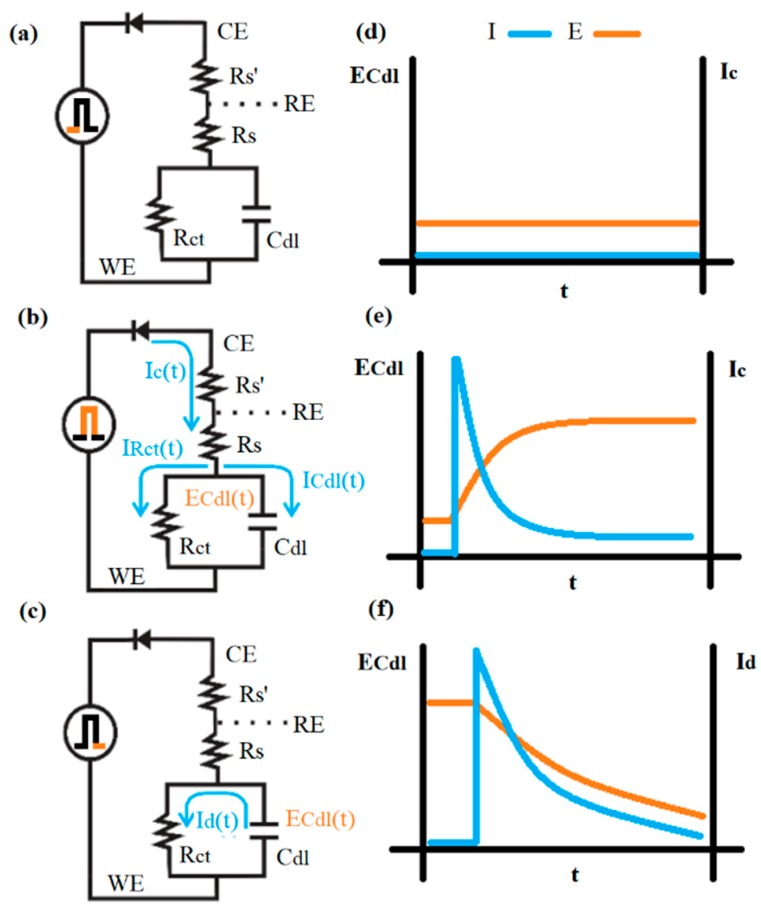
Proposed methodology to determine the potential relaxation: (**a**,**d**) equilibrium double layer; (**b**,**e**) double-layer charging; (**c**,**f**) double-layer discharging.

In this method, schematized in Figure 2, a double potential step is applied from an initial potential *E_i_* = 0 V, to a final potential *E_f_* = η, with respect to the open circuit potential value; then, after 20 ms, the potential is returned to its initial value [25]. The potential η corresponds to the formal potential of the redox couple used as an indicator, while on the other hand η is a potential value small enough that the system has a virtually linear *I-E* behavior.

In Figure 2, the working electrode (WE) corresponding to the DNA/Au system is represented by a Randles circuit, with $R_{s}$ representing the solution resistance, $C_{dl}$ as the double-layer capacitance and $R_{ct}$ as the charge transfer resistance associated with the electrochemical reaction redox indicator. This simple equivalent circuit is used here only to explain the relaxation method; a circuit that fits the real electrical behavior of the complex DNA/Au electrode is described in the results and discussion section with more precision.

At *t* = 0 (Figure 2a,d), the circuit is open because the rectifier diode is not polarized. The electrical potentials in the circuit can be expressed by

(1) $$E_{R_{s}}=E_{C_{dl}}=E_{R_{ct}}=0\;V,$$

where, $E_{R_{s}}$, $E_{C_{dl}}$ and $E_{R_{ct}}$ correspond to the rest potentials of the WE equivalent circuit elements.

At *t* > 0 (Figure 2b), the voltage η is applied to the electrode and the current flows through the circuit, with the rectifier diode said to be “turned on”(forward-bias). The instantaneous peak current is given by

(2) $$I_{0}=\frac{\eta }{R_{s}},$$

because the potential is primarily applied to *R_s_* and, thus far, no charge is resident in the capacitor. After an initial abrupt climbing, the total current of the circuit decreases as a function of time, and, simultaneously, the charging current $I_{Cdl}\left(t\right)$ of capacitor exponentially decreases (Figure 2e). Thus, the charging current of the electrode bio-interface can be expressed by

(3) $$I\left(t\right)=I_{Rct}\left(t\right)+I_{Cdl}\left(t\right),$$

In addition to the double-layer charging current, this decreasing function includes the contribution to the current of the redox reaction $I_{Rct}$. The charging current of the double-layer capacitor depends on time, according to

(4) $$I_{Cdl}\left(t\right)=I_{0}e^{-\frac{t}{\tau_{c}}},$$

where $\tau_{c}$ is the relaxing time of the capacitor charging, which for a series capacitor-resistance circuit can be expressed as

(5) $$\tau_{c}=R_{s}C_{dl},$$

Simultaneously (Figure 2e), the capacitor potential undergoes an exponential increase of the form

(6) $$E_{Cdl}\left(t\right)=\eta \left(1-e^{-\frac{t}{\tau_{c}}}\right).$$

At $t\geq 5\tau_{c}$, the double-layer charge is practically complete, then the applied potential is removed and the diode blocks the feedback current (reverse-bias) (Figure 2c). A new discharge process is observed where the opening of rectifier diode avoids the discharging of $C_{dl}$ through $R_{s}$ and thus the capacitor is only discharging through $R_{ct}$ [26]. The voltage decreases according to

(7) $$E_{Cdl}\left(t\right)=\eta e^{-\frac{t}{\tau_{d}}},$$

where $\tau_{d}$ is the relaxing time of the capacitor discharge, and its value from there to the circuit shown in Figure 2c can be calculated from

(8) $$\tau_{d}=R_{ct}C_{dl},$$

where the $\tau_{d}$ value can be estimated from the discharging curve of the ($R_{ct}C_{dl}$) parallel circuit (Figure 2f) at the time corresponding to a potential value equal to 63% of η.

Thus, the double step experiment was designed such that the electrochemical capacitors present in the complex electrode double layer could be charged in the first part of a potential step and discharged to the working electrode in the second part of pulse. As a consequence, the potential discharge provides information only of the HPV DNA sequence attached to the Au surface and their electrochemical changes, which are related to the hybridization process and associated redox reactions. The amplitude of the pulse assures a maximum charging of the HPV DNA/Au electrode while the system remains kinetically limited.

## 3. Results and Discussion

### 3.1. Step Potential

**Figure 3:**
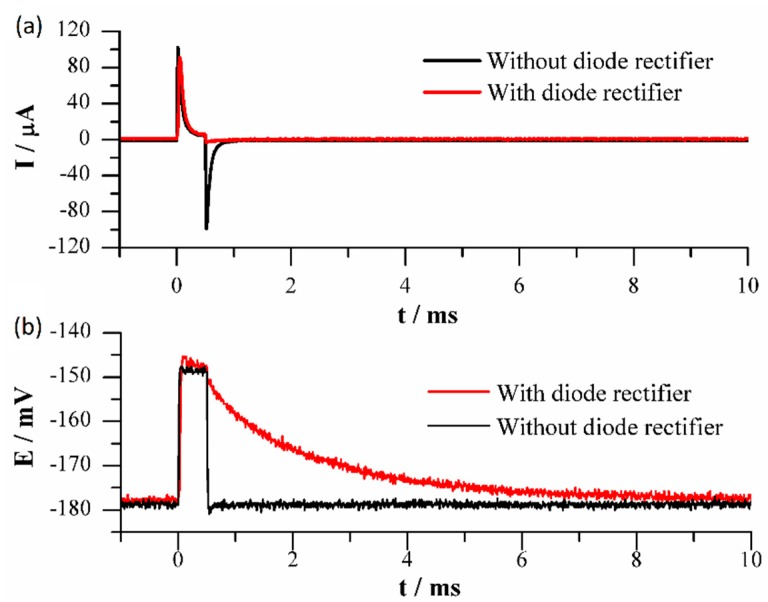
Curves of the response of ssDNA/Au electrodes to a double potential step of 30 mV. Current (**a**) and potential (**b**)

The typical I-t and E-t responses for a 30-mV potential step applied to the ssDNA/Au electrode HPV-16 are shown in Figure 3. The black line in Figure 3a corresponds to a typical double potential step chronoamperometry; in the forward step, the current spikes to a maximum value, then decays quickly to a near-zero current value by the end of the pulse period. The current flow is due to a combination of double-layer charge and faradic redox reactions at the electrode surface, including kinetic and diffusion processes. In the next stage, the applied potential is removed and the double-layer discharge of $C_{dl}$ is carried out through the resistance $R_{ct}$ and simultaneously by $R_{s}$, as they are virtually connected in parallel, corresponding to the minimal resistance path. A sharp inverse peak is also observed at the discharge step. It is important underline that this exponential decay potential $EC_{dl}$ depends only on the electrical properties of the double-layer $C_{dl}-R_{ct}$ circuit. Since the current through $R_{s}$ is zero, there will be no voltage drop across it, which as an advantage of the relaxation method in poorly conducting solutions [27].

With the same experimental conditions, a double potential step experiment was performed by using a diode rectifier connected in series with counter electrode. The I-t response for a 30 mV potential step is shown as a red line in Figure 3a. In the first step of the pulse the diode rectifier is in forward-bias condition, and a similar current peak is observed. In the discharge stage, when the applied potential is removed, the discharge of $C_{dl}$ is carried out only through the resistance $R_{ct}$, because the diode rectifier is in reverse-bias condition and is impossible for the current to flow through resistance $R_{s}$ to the potentiostat current sensor. As a result, the potential discharge curve of the double-layer capacitor can be significantly different depending on how large or small is the $R_{ct}$ value is (Figure 3b, red line). Additional information on electrode processes can be obtained if we follow the potential discharge of $C_{dl}$ measured with the reference electrode. Thus, in the backward step, the decay in potential of the double layer (Figure 3b, red line) is slower than those observed when no diode is used in the experiment (Figure 3b, black line). The decay potential curves in the backward step depend markedly on the double-layer structure, and hence can be used as a sensing principle to follow the hybridization event of DNA sequences of HPV-16, as discussed below.

Other sensors based on relaxation methods have been recently reported. Alexander et al. investigated a high-density electrochemical biosensor based on a complementary metal-oxide-semiconductor (CMOS) array [28] in which they used a coulostatic discharge rate sensing technique to detect anti-rubella and anti-mumps antibodies in human serum. However, the low currents obtained in the double-layer discharge made imperative the use of a low-leakage switch PMOS transistor and unity-gain buffer (differential amplifier) as the amplification stage in order to enable measurable potentials. In contrast with this work, a simpler electronic architecture based in a diode rectifier switching was enough to allow the discharge potential measurement in the current study.

### 3.2. Potential Relaxation Curves and DNA Equivalent Circuit Model

A closer inspection of double-layer responses revealed that the decay potential curves were in fact composed of two processes related to two different relaxation times. The decay curves of HPV-16 DNA/Au electrodes could be fit to a mathematical model represented by Equation (9) or expressed in an exponential form by the Equation (10):

(9) $$E\left(t\right)=E_{top}\left(t\right)+E_{bottom}\left(t\right),$$

(10) $$E\left(t\right)=E_{mtop}e^{\frac{-t}{\tau_{top}}}+E_{mbottom}e^{\frac{-t}{\tau_{bottom}}},$$

where E(t) is the total potential of the electrode double layer as a function of time, which includes the sum of deconvolution terms $E_{mtop}$ and $E_{mbottom}$. The exponential expressions include the relaxation times $\tau_{top}$ and $\tau_{bottom}$ related to the decay of the initial potentials $E_{mtop}$ and $E_{mbottom}$, respectively. In both equations, the potentials are expressed in respect to their open circuit potential.

**Figure 4:**
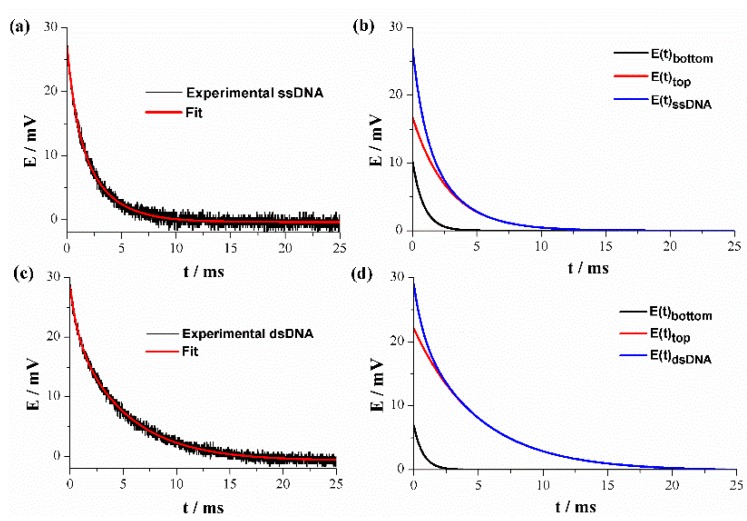
Potential step transient in ssDNA (**a**,**b**) and dsDNA (**c**,**d**) with the diode rectifier system.

The experimental E-t decay curves of HPV-16 ssDNA/Au and dsDNA/electrodes are shown in Figure 4a,c (black line), respectively, along with the fitting curves (red line) from the Equation (10). A good fit can be observed between the theoretical model and experimental data. The values of the potentials $E_{top}$ (solution/DNA) and $E_{bottom}$ (DNA/Au) of Equation (9) can be extracted from the experimental curves of the discharge of the electrode double layer. Typical discharge curves and their deconvolution components are shown in Figure 4b,d for single- and double-strand DNA, respectively, and it can be observed that $E_{top}$ is the predominant term in Equation (9) in both cases at all times. However, a higher decay time can be appreciated for the double-strand DNA than of the single-strand sequence. 

Maximum initial potential amplitudes $E_{mtop}$ and $E_{mbottom}$ are attributed to the voltage divider created by two charge transfer series resistances, $R_{ctt}$ and $R_{ctb}$, respectively. $R_{ctt}$ is the charge transfer resistance at the 3´ end of DNA. On the other side, $R_{ctb}$ is the charge transfer resistance at the DNA end attached to the Au electrode. Additionally, from the potential relaxation curves the time constants of the pair of parallel resistor-capacitor (RC) circuits related to transfer processes that happen in the two different DNA regions, next to and far from the Au electrode, can be evaluated. According to Equation (7), in the discharge or relaxation step when $E_{top}$ and $E_{bottom}$ approximate zero, time reaches values that correspond to five times the relaxation constants (*τ_top_* and *τ_bottom_*) of a series combination of two parallel RC circuits [29]. For this reason, the Randles EC that only includes a single parallel RC arrangement cannot be adequate for a comprehensive description of the electrochemical behavior of HPV-16 DNA/Au electrodes. 

**Figure 5:**
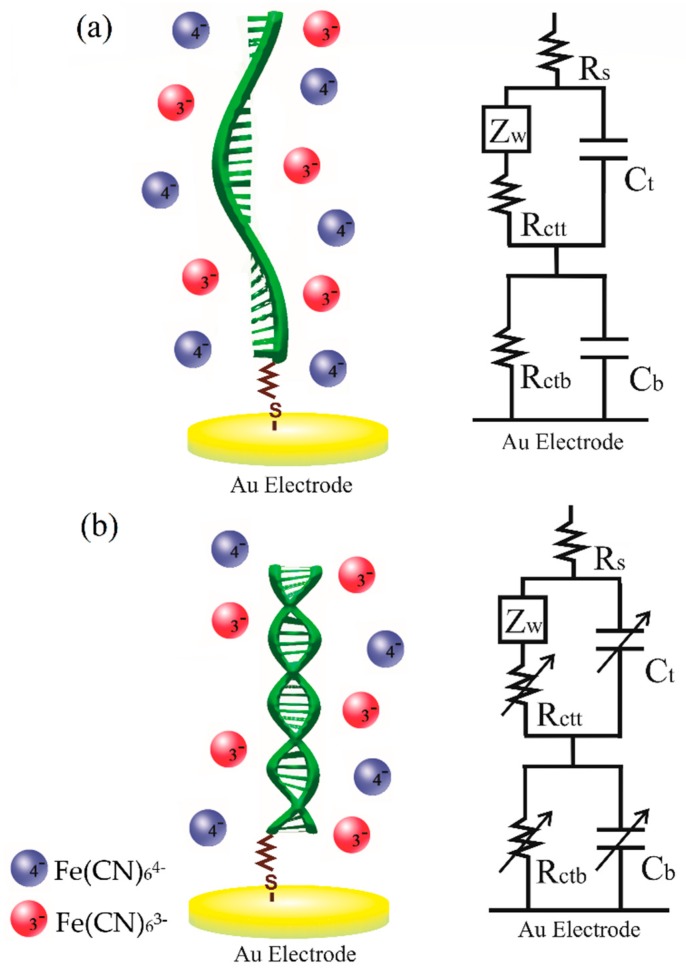
Illustration of DNA–redox couple interaction and the proposed equivalent circuit (EC): (**a**) ssDNA and (**b**) dsDNA. The arrows indicate the circuit elements that change when hybridization occurs.

An improved equivalent circuit that explains the experimental discharge behavior of DNA electrodes in a better way and takes into account the above arguments and parameters involved is proposed and presented in Figure 5. This model includes two closed loops that imply two relaxation processes. The top loop is associated with the DNA region far from the metallic electrode, and includes a Warburg impedance element ($Z_{W}$) that describes the diffusion of the redox indicator anions from the solution to DNA electrode along with the charge transfer resistance $R_{ctt}$ in parallel with the capacitor $C_{t}$. The bottom loop includes only the resistance $R_{ctb}$ and capacitor $C_{b}$ related to the electrochemical process occurring in the DNA region next to the Au electrode. $R_{s}$ corresponds to the solution resistance. This model was further proven via EIS measurements, as described later. 

By analyzing the voltages $E_{mtop}$ and $E_{mbottom}$ (see Figure 4b,d), it can be seen that the potential $E_{mtop}$ increases while $E_{mbottom}$ decreases slightly with hybridization. This behavior can be attributed to an $R_{ctt}$ increase, whereas the resistance $R_{ctb}$ remains practically constant. The increase in $R_{ctt}$ can be explained by a higher repulsion of the redox indicator anions due to the addition of phosphate groups when the double-stranded DNA is formed, and this anionic removal hinders the electron transfer process. Likewise, the increase in $R_{ctt}$ can be verified by the greater relaxation time observed in curve $E_{mtop}$ of Figure 4d in respect to the relaxation time before hybridization (Figure 4b).

A summary of typical parameter values obtained from the deconvolution of discharge curves of DNA electrodes before and after hybridization are presented in Table 2. The figures shown in the table correspond to the mean values ± RSD of three different biosensors fabricated in the same way. It can be observed that the highest percentage changes correspond to $E_{mtop}$ and $\tau_{top}$ parameters. In this way, the top peak-potential and relaxation time are the most adequate properties to be used as analytical variables for HPV-16 DNA sensing. 

**Figure 6:**
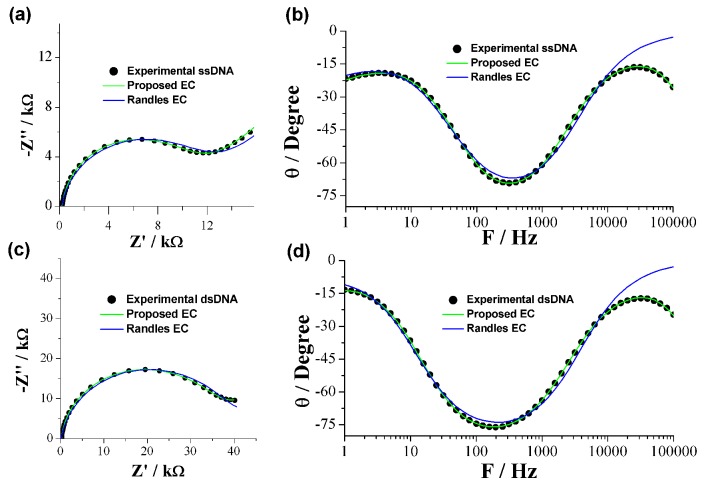
EIS Nyquist and Bode plots of the HPV-16 DNA/Au electrode. (**a**,**b**) ssDNA, (**c**,**d**) dsDNA.

In order to corroborate the proposed equivalent circuit, EIS measurements were done on modified electrodes before and after hybridization, and data obtained were fit to EC by CNLS. For comparison purposes, the data were also fit to a simple Randles circuit. In Figure 6 the Nyquist and Bode plots of experimental data are shown, along with the lines that correspond to the fit of each model. In all graphics across the entire frequency range a better fit can be observed for the proposed EC than the Randles circuit. On the other hand, a poor fit of the Randles EC is observed in the low-frequency range in Nyquist plots (Figure 6a,c), and in the high-frequency range in the Bode plots (Figure 6b,d). The results confirm that the proposed EC can be used to describe the double-layer electrochemical behavior of the DNA/Au electrode in a comprehensive way.

### 3.3. Analytical Performance of the DNA Biosensor

**Figure 7:**
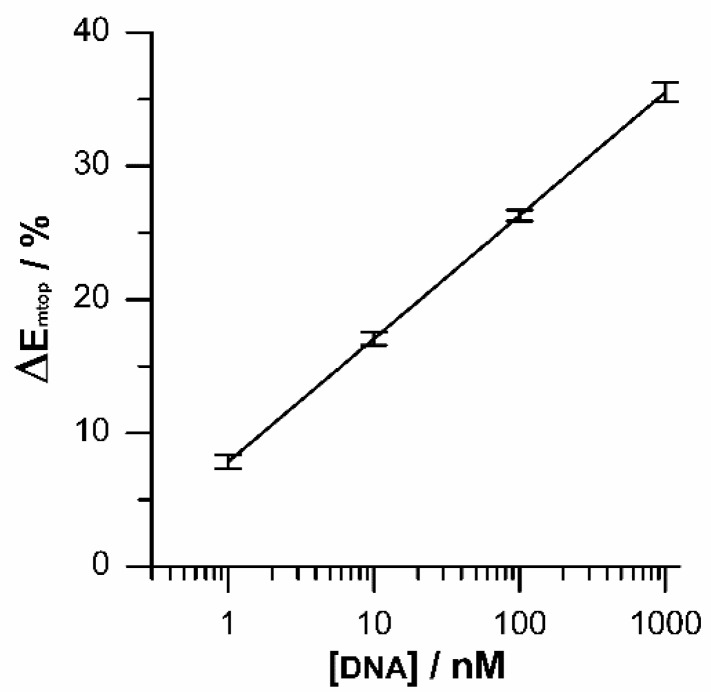
Corresponding $E_{top}$ peak voltages vs different target DNA concentration.

With the aim of evaluate the analytical application of DNA/Au electrodes for HPV-16 DNA sensing, the increase in the $E_{mtop}$ was measured for hybridization with a wide concentration range from 1 nM to 1 μM of complementary DNA under optimized conditions. Figure 7 shows the calibration curve that relates the percentage increase in peak potential ($\Delta E_{mtop}$) to the concentration. A good linearity can be observed in the graph throughout the complete concentration range. Furthermore, the small size of deviation bars indicates a good repeatability of the method. The linear regression equation was $\Delta E_{mtop}$ = 4.33 + 4.941 log ($C_{\mathrm{DNA}}$/nM), and a squared linear correlation coefficient of 0.991 was obtained with a detection limit of 0.38 nM. These results are evidence that the potential relaxation method here proposed is feasible to detect HPV-16 DNA in nanomolar concentration.

**Figure 8:**
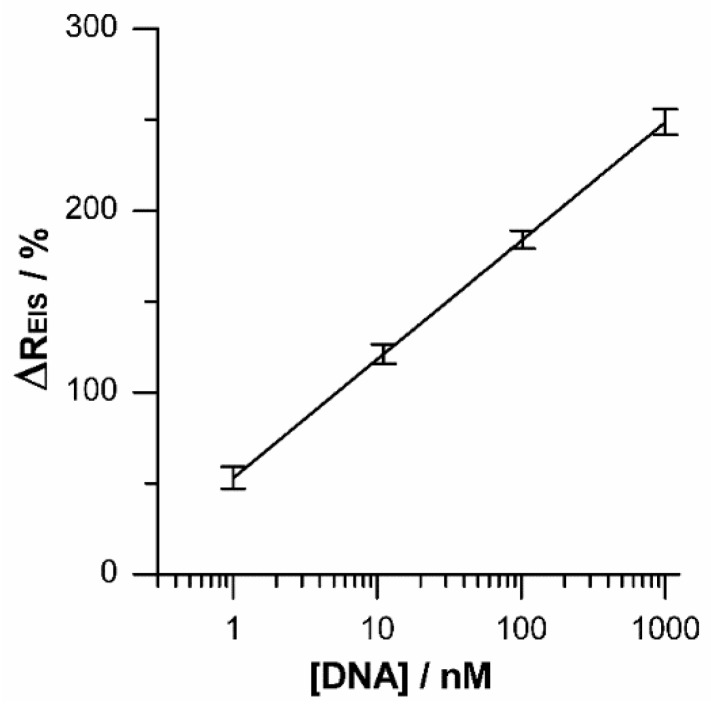
The logarithm relationship between $\Delta R_{EIS}$ and target DNA concentration with the new equivalent circuit.

In order to compare the analytical results previously described, the traditional EIS technique was employed to detect the hybridization. For this purpose, the change in global charge transfer resistance ($\Delta R_{EIS}$) was evaluated as a DNA concentration function by using the proposed equivalent circuit. It can be seen from Figure 8, that the resistance change was a linear function of the logarithm of the DNA concentration in the same range from 1 nM to 1 μM. In this case the linear regression equation was $\Delta R_{EIS}$ = 53.042 + 28.33 log ($C_{\mathrm{DNA}}$/nM) with a R^2^ = 0.987 and a detection limit of 0.26 nM. The limits of detection for both methods were similar. EIS showed a better sensitivity to DNA detection although the data dispersion was larger than relaxation measurements. It is important to emphasize that EIS requires a lot of time and complex electronic devices to obtain a complete spectrum acquisition, while a potential relaxation experiment takes only 25 ms to complete using a simple electronic system.

### 3.4. Specificity of the DNA Biosensor

**Figure 9:**
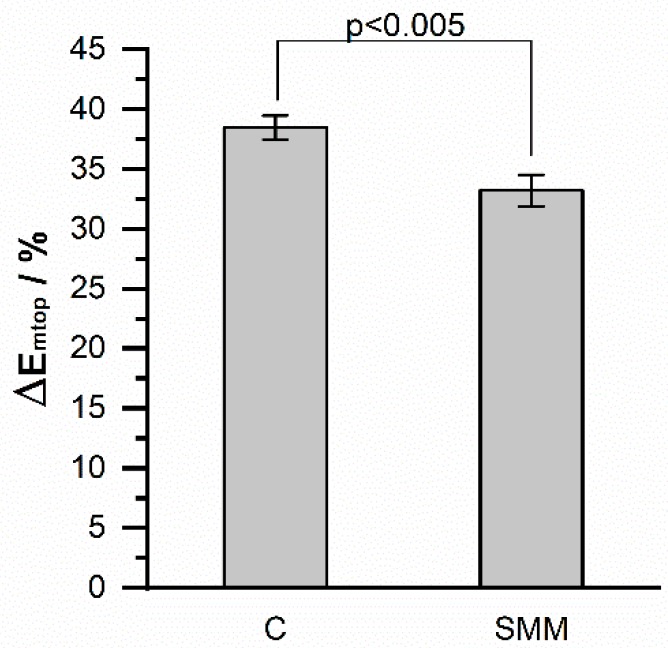
Comparative graph of the $\Delta E_{mtop}$ of the biosensor response to different DNA sequences. Complementary DNA (C) and single-base mismatch (SMM).

The specificity of the biosensor was evaluated by measuring the increase in the maximum potential due to the hybridization of 1 μM of the complementary sequence (C), or for a single-base mismatch (SMM), both of which are shown in Table 1. The graph shown in Figure 9 corresponds to the mean of three independent experiments where the error bars are the standard deviations. As can be seen, the complementary sequence reached a value of 38.47% that was significantly greater than the 33.2% value obtained with the single-base mismatched sequence. This means that the relaxation method is highly specific, and allows the detection of even a single base change in the oligonucleotide sequence.

Even though an acceptable performance and selectivity have been obtained for this sensor in the analysis of synthetic HPV-16 DNA, further research is needed before applying this method in complex biological samples such as serum or vaginal exudate. A pre-treatment of samples is required in order to extract and purify the viral DNA before hybridization can be detected by the double-layer relaxation approach. The use of enzymatic techniques to cut the DNA in small fragments is required, as well as biochemical procedures to obtain the appropriate samples for electrochemical sensing. 

On the other hand, improvement of the sensitivity and versatility of relaxation sensors can be obtained by changing the type of electrode used, and in this sense the employment of interdigitated electrodes can provide the simultaneous detection of different HPV types as well as the screening of several viruses. Recently, the use of microcantilevers has been reported for sensing biomolecules, including DNA structures [30,31]. Microcantilever arrays are compatible with electrochemical measurements, and the combination of double-layer relaxation and cantilever deformation variations could make the design of high sensitivity biosensors feasible.

## 4. Conclusions

In this work, it has been proven that electrochemical changes due to double-layer relaxation on electrodes modified with DNA can be useful for the development of an HPV-16 DNA biosensor. The relaxation variables following the hybridization process were change in maximum potential and relaxation time. In this work, the parameter $\Delta E_{mtop}$ was selected because it facilitates the signal amplification process necessary for the development of a practical device. Further information on double layers can be extracted from the complete relaxation curves, which can describe kinetics and diffusional processes for a more comprehensive description of the charge-transfer mechanism in DNA electrodes. 

In addition, an equivalent circuit was presented that describes the potential relaxation of the double layer of a DNA-modified electrode and enables extraction by deconvoluting the parameters for HPV-16 DNA detection. EIS measurements indicated that the proposed equivalent circuit showed an improved data-fitting compared to the Randles model. 

The calibration curves obtained indicate that the double-layer relaxation method allows a detection limit to be reached in the order of 10^−10^ M of the HPV complementary oligonucleotide in short detection times close to 25 ms. The high selectivity of the biosensor enabled discrimination between complementary and single mismatch sequences.

The presented results suggest that a biosensor based on double-layer discharge has the potential to develop an easy-to-construct device for HPV-16 detection.

## Figures and Tables

**Table 1:** DNA sequences used in this study.

Oligonucleotide	Sequence
Probe	5′-HS(CH2)_6_GTCATTATGTGCTGCCATATCTACTT-CAGA-3’
Complementary	5´-TCTGAAGTAGATATGGCAGCACATAATGAC-3´,
Single-base mismatch	5´ TCTGAAATAGATATGGCAGCACATAATGAC-3´

**Table 2:** Parameters obtained from the fitting for the reverse transient in ssDNA and dsDNA HPV-16.

Parameter	ssDNA Value	ssDNA-dsDNA Difference Δ %
$E_{OC}$	−178.06 ± 0.19	1.76 ± 0.1
$E_{mtop}$	16.86 ± 0.25	31.85 ± 1.15
$\tau_{top}$	2.82 ± 0.33	78.72 ± 2.83
$E_{mbottom}$	10.43 ± 0.15	25.02 ± 1.13
$\tau_{bottom}$	0.909 ± 0.01	18.81 ± 1.61

Note: *E* is expressed as mV and *τ* in ms.
